# Assessment of knowledge, attitude and practice toward COVID-19 and associated factors among health care workers in Silte Zone, Southern Ethiopia

**DOI:** 10.1371/journal.pone.0257058

**Published:** 2021-10-05

**Authors:** Mubarek Yesse, Mohammed Muze, Shemsu Kedir, Bedru Argaw, Mohammed Dengo, Tajudin Nesre, Faris Hamdalla, Awol Saliha, Tofik Mussa, India Kasim, Abdulfeta Kedir, Tofik Delebo, Awol Sunkemo, Yesuf Badeg, Dureti Ensarmu, Dereje Abebe, Amara Dessalegn, Henok Ayelign

**Affiliations:** 1 Department of Nursing, College of Medicine and Health Sciences, Werabe University, Awasa, Southern Ethiopia; 2 Department of Statistics, College of Natural and Computational Science, Werabe University, Awasa, Southern Ethiopia; 3 Department of Plant Science, College of Agriculture, Werabe University, Awasa, Southern Ethiopia; 4 Department of Siltigna, College of Humanity Sciences, Werabe University, Awasa, Southern Ethiopia; 5 Department of English, College of Humanity Sciences, Werabe University, Awasa, Southern Ethiopia; 6 Department of Biology, College of Natural and Computational Science, Werabe University, Awasa, Southern Ethiopia; China University of Mining and Technology, CHINA

## Abstract

**Introduction:**

COVID-19 is a pandemic respiratory disease caused by the highly contagious novel coronavirus (SARS-CoV 2). The disease is now quickly spreading around the world, resulting in ongoing coronavirus pandemic. Healthcare workers are more susceptible to COVID-19 infection than the general population due to frequent contact with infected individuals.

**Objective:**

This study was aimed to assess knowledge, attitude and practice towards COVID-19 and associated factors among health care workers.

**Methods:**

Facility-based cross-sectional study design was conducted among health care workers in Silte Zone. A total of 379 health professionals were selected using multistage stratified sampling technique. Self-administered questionnaire was used to collect data. Binary logistic regression model was used to see association between outcome and independent variables.

**Results:**

This study found 74.9%, 84.2% and 68.9% prevalence of adequate knowledge, positive attitude and good practice respectively. Working in comprehensive specialized hospital (AOR = 4.46, 95% CI = 1.46–13.62).having MSC degree (AOR = 10.26, 95% CI = 2.27–46.44), and training on COVID-19 (AOR = 6.59, 95% CI = 2.97–14.65) were strongly associated with knowledge of health care workers. On the other hand, older age (AOR = 3.35, 95% CI = 1.07–10.50), training on COVID-19 (AOR = 3.73, 95% CI = 1.82–7.63), Work experience (AOR = 3.78, 95% CI = 1.46–9.80) and Knowledge (AOR = 5.45, 95% CI = 2.60–11.43) were significantly associated with attitude, whereas source of information from friends or colleagues (AOR = 3.13, 95% CI = 1.28–7.66), working in primary hospital (AOR = 0.36, 95% CI = 0.21–0.620) and having good knowledge (AOR = 1.80, 95% CI = 1.03–3.14) were strongly associated with good practice of health care workers.

**Conclusion:**

This study found majority of health care workers had good level of knowledge and positive attitude toward COVID-19, but lower proportion of health care workers practices sufficiently in the study area. Type of health facilities, level of education, training on COVID-19, work experience, type of source of information were significantly associated with knowledge, attitude and practice of health care workers. Stakeholders need to focus on interventions that increase preventive practices of health care workers.

## Introduction

COVID-19 is an emerging respiratory disease caused by the highly contagious novel coronavirus (SARS-CoV 2) [1]. The disease was first identified in December 2019 in Wuhan, Hubei province of China, and currently quickly spreading around the world, resulting in the ongoing coronavirus pandemic [2]. As of 5^th^ January 2020, globally more than 78.8 million cases have been reported across 218 countries and territories, resulting in more than 1,733,488 deaths. And more than 55,452,004 people have recovered [3].

Ethiopia confirmed first case of COVID-19 at 13 March 2020. Person found positive is a 48 year old Japanese man who came to Ethiopia on March 4, 2020 from Burkina Faso. Since then, number of cases is increasing. As of 5th January 2021, Ethiopia has recorded more than 125622 positive cases involving 1948 deaths [4]. Apart from personal health, COVID-19 also affects economy of the countries. According to the estimation of the United Nations Economic Commission for Africa, COVID-19 will shave 2.9 percentage points off of Ethiopia’s economic growth for fiscal year 2020 and the pandemic will also affect the Ethiopia’s export significantly [5].

Majority people infected with the COVID-19 virus will have mild to moderate respiratory illness and recover without requiring special treatment. Older people and those with underlying medical problems such as diabetes, cardiovascular disease, chronic respiratory disease, and cancer are extra likely to develop severe illness. The virus spreads primarily through droplets of saliva or discharge from the nose when an infected person sneezes or coughs [6–8]. The mortality rate of this infection is about 3–6% but, according to some authors, this percentage may be seriously underestimated [9].

Health care workers (HCWs) are more susceptible to COVID-19 infection than the general population due to frequent contact with infected individuals. Additionally, some procedures such as non-invasive ventilation, high-flow nasal cannula and bag-mask ventilation may generate higher aerosol volumes leads to increased risk of infection [10, 11]. The longer working hours (due to the increased number of infected people in hospital) also put them at risk of infection[12]. Number of studies reported various prevalence rate of COVID-2019 among HCWs 9.8% in New york [13] and 10.1% from a systemic review of developed countries [14].

Protection of HCWs and inhibition of intra-hospital transmission of COVID-2019 are important parts of epidemic response and this requires that HCWs must have updated knowledge regarding COVID-2019 [15]. Literature suggests that lack of knowledge and misunderstandings among HCWs leads to delayed diagnosis, spread of disease and poor infection prevention practice [16, 17]. Currently, there is scarce information regarding knowledge, attitude and practice of HCWs towards COVID-19 in Ethiopia particularly in southern part. Furthermore, previous studies in other study areas found risk factors for poor preventive practice of HCWs [18–20]. The risk factors may not be the same across geographic locations. Therefore, the present study was aimed to determine current status of knowledge, attitude and practice) (KAP) towards COVID-19 and associated factors among HCWS in the study area.

## Materials and methods

### Study area, design and subjects

Facility-based cross-sectional study design was conducted from June 1 to July 2020 in Silte Zone. The Silte Zone is one of the Zones of the Ethiopian Southern Nations, Nationalities and Peoples Region (SNNPR) and found 172 km away from the capital city, Addis Ababa. This zone consists of 3 administration towns, 10 rural weredas and 212 kebeles. Based on last Census conducted by central statistical agency of Ethiopia (CSA), in 2018 this Zone has estimated population of 1,017,557. The Zone currently has four hospitals and 33 health centers. All HCWs working in the health facilities were source population of this study. Selected HCWs who full fill inclusion criteria were the study population. HCWs age 18 years old and above were included in the study. Sample size was calculated using single population proportion formula based on the following assumptions; 50% prevalence (P), 95% confidence level and margin of error of 5%. By applying the finite population correction formula and adding 10% non-response rate, the sample size was 391. To select study participants, First HCWs were stratified based on the type of health facilities (health center, primary hospital and comprehensive specialized hospital). Eleven health centers, one primary hospital and one compressive specialized hospital were randomly selected from each stratum. Sample size was allocated to health facilities proportionately. Accordingly, 202, 120 and 69 study participants were allocated to health centers, primary hospitals and comprehensive specialized hospital respectively. Systematic sampling technique was used to select study subjects. Total of 1871 HCWs were found in selected health facilities. Therefore, K was calculated as 5(1871/391) and from the first five HCWs, the 2nd HCW was randomly selected by using a lottery method. Accordingly, every 5th HCWs were selected based on their availability in health facilities.

### Measurements and data collection tools

The structured questionnaire was prepared after reviewing published literatures [21–25]. The questionnaire contains items on socio-demographic and economic factors, Knowledge related items, attitude related items and practice related items. Knowledge section comprised of 30 items assessed nature of disease, etiology, symptoms, risk group, testing, transmission, treatment and precautions/preventions. Each item was responded as yes, no or I don’t know. The right answer was labeled as 1 while wrong answer was labeled as 0. Total score ranges from 0–30 and a cut off level of <17 was set for poor knowledge and ≥18 (60% and above) for good knowledge. Attitude section comprised of 26 items assessing attitude of healthcare workers toward treatment, infection control procedure and information regarding COVID-19. Response of each item was recorded on 5-point Likert scale as follows strongly agree (5-point), agree (4-point), Undecided (3-point), disagree (2-point), and strongly disagree (1-point). Total score ranges from 26 to 130, with score of >78 (>60%) indicates positive attitude toward COVID-19 [21–25]. Practice section included 16 items regarding use of face mask, and practice of other precautionary measures. Each item was responded as yes (1-point) and (0-point). Practice items total score ranged as 0–16, and a score of ≥10 demonstrated good practice and a score of <10 indicates poor practice toward precautionary measures of COVID-19 [21–25].

### Data quality control, processing and analysis

Data quality was assured by caring out careful design of the questionnaire, appropriate recruitment of data collectors and by giving adequate training and follow-up for data collectors and supervisors. The questionnaire was pre-tested on 5% of HCWs from health facilities not included in actual studies and modified before the main study began. The data were checked for completeness and consistency and then coded, entered and stored into the computer using Epi-data software. Data was exported to SPSS version 21 statistical packages for analysis. Descriptive statistics were calculated for demographic and economic factors, information related and job related factors. Binary logistic regression analysis was applied. All predictor variables that have an association in bivariable analysis with p-value < 0.25 were entered into multivariable logistic regression model. In multivariable logistic regression analysis, those variables with a p-value ≤ 0.05 were considered as statistically significant.

### Ethical considerations

Ethical clearance was obtained from Werabe University before conducting the study. At the time of data collection, written consent was obtained from the participants. Each participant was requested to sign it to certify that he or she had agreed freely to participate in the study. Those not willing to participate were given the right to do so. Confidentiality of responses was also ensured throughout the research process.

## Results

### Socio-demographic characteristics of respondents

Total of 379 HCWs were participated in this study, making response rate of 96.9% were included. The mean age of the participants was 33.46 (± 6.43). Majority (87.3%) of the respondents were rural dwellers. Regarding educational level of participants, 44.9% of the respondents had first degree level of education. About 43% were married, 52.0% of the participants work at health centers and 38.3% of the participants were nurses. About one third of the respondents get information about COVID-19 mainly from TV and mean monthly income of the participant was 8192.7 (±9284.0) (Table 1).

**Table 1:** Socioeconomic and demographic characteristics of the study participants, Silte zone, southern Ethiopia, 2020.

Variables (n = 379)	Category	Frequency	Percent
**Age**	20–30 Years	74	19.5
	31–40 Years	259	68.3
	> = 41 Years	46	12.1
**Sex**	Male	223	58.8
	Female	156	41.2
**Marital status**	Married	163	43.0
	Unmarried	159	42.0
	Separated	57	15.0
**Religion**	Muslim	298	78.6
	Orthodox	29	7.7
	Protestant	52	13.7
**Residence**	Urban	331	87.3
	Rural	48	12.7
**Type of health institution**	Health center	197	52.0
	Primary hospital	115	30.3
	Comprehensive Specialized hospital	67	17.7
**Level of education**	Diploma	184	48.5
	Degree	170	44.9
	Masters	25	6.6
**Source of information**	TV	116	30.6
	Social Media	110	29.0
	FMoH	72	19.0
	Friends/ colleagues	55	14.5
	Radio	26	6.9
**Profession**	Nurse	145	38.3
	Midwife	83	21.9
	Laboratory	32	8.4
	Public health	31	8.2
	Pharmacy	40	10.6
	Anesthesia	13	3.4
	Physician	22	5.8
	Others	13	3.4
**Training**	Yes	124	32.7
	No	255	67.3
**Working hour/day**	< = 8 hour	119	31.4
	> 8 hour	260	68.6
**Monthly family income**	< = 5000 Birr	193	50.9
	5001–10000	139	36.7
	> 10000	47	12.4
**Work Experience**	< = 5 Years	134	35.4
	6–10 Years	173	45.6
	> 10 Years	72	19.0

### Knowledge of HCWs

Of the surveyed HCWs, 75% (95% CI = 70.2%-79.2%) of the HCWs had demonstrated adequate knowledge of COVID 19. In multivariate analysis, type of health institution, level of education, and training on COVID-19 were strongly associated with knowledge of HCWs about COVID-19. HCWs of comprehensive specialized hospital had good knowledge of COVID-19 4.46 times more likely than health centers (AOR = 4.46, 95% CI = 1.46–13.62). HCWS who received training on COVID-19 were 6 times had good knowledge of COVID-19 than health centers than who didn’t receive the training (AOR = 6.59, 95% CI = 2.97–14.65). Level of education also significantly associated with the knowledge. Having BSC degree (AOR = 2.84, 95% CI = 1.56–5.12) and MSC degree positively associated with of knowledge HCWs about COVID-19 (Table 2).

**Table 2:** Factors associated with Knowledge of health care workers about COVID-19 in Silte zone, southern Ethiopia, 2020.

Variables	Knowledge Status		Odds Ratio at 95% CI		P value
	Good	Poor	COR (95% CI)	AOR (95% CI)	
	No. (%)	No. (%)			
**Age in years**					
20–30 Years	188	73	1		
31–40 Years	58	14	0.62 (0.33–1.18)	0.60 (0.29–1.23)	0.162
> = 41 Years	38	8	1.15 (0.44–3.0)	1.30 (0.46–3.73)	0.622
**Type of health institution**					
Health center	131	66	1		
Primary hospital	90	25	1.81 (1.10–3.10)	1.78 (0.99–3.21)	0.054
Comprehensive hospital	63	4	7.95 (2.77–22.75)	4.46 (1.46–13.62)	0.009
**Level of education**					
Diploma	113	71	1		
Degree	148	22	4.22 (2.47–7.23)	2.84 (1.56–5.12)	0.001
Masters	23	2	7.23 (1.65–31.59)	10.26 (2.27–46.44)	0.003
**Training on COVID-19**					
No	168 (59.2)	87 (91.6)	1	1	
Yes	116 (40.8)	8 (8.4)	7.51 (3.51–16.09)	6.59 (2.97–14.65)	0.000
**Monthly income**					
2000–5000 birr	130	63	1		
5001–10000 birr	117	22	1.79 (0.84–3.84)	1.72 (0.93–3.20)	0.085
> 10000 birr	37	10	2.58 (1.49–4.45)	1.75 (0.76–4.05)	0.192

### Attitude of HCWs

In this study, 319 (84.2%) (CI = 80.2%-87.6%) of HCWs had demonstrated positive attitude. In multivariate analysis, age of the HCWs, training on COVID-19, Work experience, and Knowledge on COVID-19 showed significant association with attitude of HCWs toward COVID-19. Age between 31–40 Years (AOR = 2.92, 95% CI = 1.44–5.92) and ≥41 Years old of HCWs (AOR = 3.35, 95% CI = 1.07–10.50) were significantly associates with positive attitude. Similarly, HCWS who received training on COVID-19 were 3.73 times had positive attitude to COVID-19 than who didn’t received the training. Work experience between 6–10 Years (AOR = 2.20, 95% CI = 1.15–4.22) and > 10 Years (AOR = 3.78, 95% CI = 1.46–9.80) were positively associated with the attitudes of the HCWs. Knowledge on COVID-19 also significantly associated with positive attitude (AOR = 5.45, 95% CI = 2.60–11.43) (Table 3).

**Table 3:** Factors associated with attitude of HCWs toward COVID-19 in Silte zone, southern Ethiopia, 2020.

Variables	Attitude Status		Odds Ratio at 95% CI		P value
	Positive	Negative	COR (95% CI)	AOR (95% CI)	
	No. (%)	No. (%)			
**Age in years**					
20–30 Years	53	19	1	1	
31–40 Years	225	36	2.24 (1.19–4.21)	2.92 (1.44–5.92)	.003
> = 41 Years	41	5	2.94 (1.01–8.54)	3.35 (1.07–10.50)	.038
**Type of health institution**					
Health center	87	28	1		
Primary hospital	172	25	0.45 (0.25–0.82)	0.37 (0.19–0.71)	0.003
Comprehensive hospital	60	7	1.25 (0.51–3.03)	1.30 (0.49–3.44)	0.601
**Training on COVID-19**					
No	221 (59.2)	34 (91.6)	1		
Yes	98 (40.8)	24 (8.4)	1.72 (0.98–3.03)	3.73 (1.82–7.63)	0.000
**Work Experience**					
1–5 Years	102	32	1		
6–10 Years	152	21	2.27 (1.24–4.16)	2.20 (1.15–4.22)	.017
> 10 Years	65	7	2.91 (1.21–6.99)	3.78 (1.46–9.80)	.006
**Knowledge about COVID-19**					
Poor Knowledge	69	26	1		
Good knowledge	250	34	2.77 (1.56–4.93)	5.45 (2.60–11.43)	0.000

**Note:** Model classification accuracy = 85.5, Hosmer and Lemeshow = 0.269, Nagelkerke R = 226

### Preventive practices of HCWs

About 68.9% (CI = 64.1%-73.4%) of the HCWs had implemented appropriate preventive practice of COVID-19. In the multivariate model, source of information, type of health institution and knowledge of COVID-19 are strongly associated with preventive practice of health care workers. HCW’s source of information from friends/colleagues were 3 times more likely had good preventive practice than source of information from electronic information (AOR = 3.13, 95% CI = 1.28–7.66). HCWs of primary hospital were less likely had good practice than health centers AOR = 0.36 95% CI = 0.21–0.620). knowledge of COVID-19 also showed significant association with good preventive practices of HCWs. HCWs who had good knowledge on COVID-19 were 2 times more likely implement good preventive practice than who had poor knowledge AOR = 1.80, 95% CI = 1.03–3.14) (Table 4).

**Table 4:** Factors associated with preventive practice of COVID-19 among health care workers in Silte zone, southern Ethiopia, 2020.

Variables	Practice status		Odds Ratio at 95% CI		P Value
	Good	Poor	COR (95% CI)	AOR (95% CI)	
	No. (%)	No. (%)			
**Age in years**					
20–30 Years	53	19	1	1	1
31–40 Years	225	36	1.61 (0.93–2.77)	174 (0.95–3.19)	0.072
> = 41 Years	41	5	1.93 (0.55–2.58)	1.03 (0.44–2.39)	0.952
**Sex**					
Male	147	76	1		1
Female	114	42	1.40 (0.90–2.20)	1.62 (0.98–2.67)	0.058
**Marital status**					
Married	117	46	1.37 (0.73–2.57)	0.80 (0.47–1.35)	1
Unmarried	109	50	1.60 (0.85–3.01)	0.58 (0.29–1.16)	0.401
Separated	35	22	1		0.126
**Source of information**					
TV	66	44			
Social media	49	23	0.68 (0.39–1.17)	0.74 (0.41–1.33)	0.312
FMoH website	80	36	0.96 (0.51–1.81)	0.96 (0.49–1.89)	0.911
Friends/relatives	19	7	2.64 (1.13–3.16)	3.13 (1.28–7.66)	0.013
Radio	47	8	1.22 (0.47–3.16)	1.38 (0.50–3.81)	0.540
**Type of health institution**					
Health center	61	54	1		1
Primary hospital	149	48	0.36(0.22–0.59)	0.36 (0.21–0.620)	0.000
Comprehensive hospital	51	16	1.03 (0.54–1.97)	1.09 (0.54–2.20)	0.821
**Average working hour**					
< = 8 hours	73	46	1		1
> 8 hours	188	72	1.65 (1.04–2.60)	1.36 (0.82–2.60)	0.233
**Knowledge about COVID-19**					
Poor Knowledge	57	38	1		1
Good knowledge	204	80	1.70(1.05–2.76)	1.80 (1.03–3.14)	0.039
**Attitude towards COVID-19**					
Negative	33	27			1
Positive	228	91	2.05 (1.17–3.60)	1.43 (0.76–2.69)	0.267

**Note:** Model classification accuracy = 72.8, Hosmer and Lemeshow = 0.899, Nagelkerke R = 1.71

## Discussion

This study was conducted to assess knowledge, attitude and practice towards COVID-19 and associated factors among HCWs in health facilities. The outputs of this study are essential to HCWs, health facilities, health management authorities to mitigate the spread of COVID-19 [26]. According to this study, 74.9% % of HCWs had demonstrated good knowledge on COVID-19. The result is comparable with other two studies conducted in Ethiopia where 70% [18] and 73.8% [27] of HCWs had sufficient knowledge. Nearly comparable finding is also reported in a study conducted in Uganda where 69% of HCWs had adequate knowledge of COVID-19 [19]. However, it is lower than findings reported in China where 89% [20], and in Pakistan 93.2% [24]. On the other hand, the finding is higher than studies conducted in Saudi Arabia and India where 45% [28] and 54.7% of HCWs had good knowledge respectively [29]. This difference might be related to variations in study area. Battling pandemic across countries may not be the same leads to deference in knowledge. Another possible reason is that it could be due to differences in the cut-off points used to categorize knowledge. In this survey, Type of health institution, level of education, and training on COVID-19 were strongly associated with knowledge of health care workers about COVID-19.

HCWs of Comprehensive specialized hospital were about 4.46 times more likely had good knowledge of COVID-19 than health centers. This may be explained by HCWs working in comprehensive specialized hospital tend to have higher level of education this may leads to deference in knowledge. HCWS who received training on COVID-19 6 times had good knowledge of COVID-19 than who didn’t receive the training.

Also level of education having BSC degree and MSC degree positively associated with the level of knowledge. Some studies have reported that educational level of HCWs had strongly associated with level of knowledge on COVID-19 [27].

Our study also found that 84.2% of HCWs had a positive attitude toward COVID -19. This finding was comparable to a study conducted in Pakistan where 86.5% of HCWs had positive attitude toward COVID-19 [28]. However, this finding was higher than other findings such as 65.7% positive attitude toward COVID-19 from northern Ethiopia [27], 53.4% from Nepal [30] and 21% from Uganda [19]. The possible reason for the difference might be due to the study setting and period; our study covers both health centers and hospitals whereas the previous was only hospital based. Other reason to the variation might be caused by differences in number of questions used and cut off value used to categorize the attitude. Increased Age of the HCWs was positively associates with positive attitude. This is in line with finding of a study done in Nepal [30]. The higher age, the longer is the experience in dealing with emergencies, ultimately showing confidence and optimism [31]. Therefore, increasing age could be the reason for a positive attitude toward COVID-19. Training on COVID-19 also demonstrated positive association with attitude. HCWS who received training on COVID-19 3.73 times had positive attitude to COVID-19 than who didn’t received the training. Training could increase knowledge leads to reduction of misinformation. Hence, training could be the reason for a positive attitude toward COVID-19. Increased work experience of HCWs significantly associated with the positive attitude toward COVID-19. This was supported by previous studies [20]. Knowledge on COVID-19 showed significant association with their attitude toward COVID-19. This finding is in congruent with previous studies [27]. Knowledge is a prerequisite for establishing beliefs, forming positive attitudes so that knowledge modulates the attitude [31].

In our study, bout 68.9% of the HCWs implemented appropriate practice. This finding was nearly comparable with studies conducted in Pakistan [29] and Uganda [19] where 73.4%, and 74% of HCWs had implemented good practice respectively. However, higher good practice 88.7% in Pakistan [28], and 81.5% in Nepal [30] were reported. Good preventive practice of the HCWs was significantly associated with source of information of COVID-19. HCW’s source of information from friends/colleagues 3 times more likely had good preventive practice than source of information from electronics. The possible reason for the association might be due to the study setting. Majority of the current study area is rural. Information from electronics (TV/Radio) no easily accessible in rural area may leads to none association with preventive practice of HCWs. HCWs of primary hospital were less likely had good practice than health centers. This is congruent with previous studies [18]. Level of knowledge also shows significant association with good practices of HCWs. HCWs who had good knowledge on COVID-19 about 2 times more likely implement good preventive practice than who had poor knowledge. Similar findings were reported in previous studies [18, 28]. Knowledge of a disease may influences practices of individuals [32].

This study has some limitations that might have minimal impact on the study findings. It is a cross-sectional survey, so we could not assess the changes, causes and effect relationship. Furthermore, there is a possibility of information bias, as surveys were self-administered. Despite these limitations, it is a survey on the KAP includes all public health facilities so that the finding is generalizable to public health facilities of the study area. This survey would probably provide up-to-date information and improve preventive practice of HCWs.

## Conclusion

This study found majority of HCWs had good level of knowledge and positive attitude toward COVID-19, but lower proportion of HCWs practices sufficiently in compare to magnitude of good knowledge and positive attitude. Type of health facilities, level of education, training on COVID, work experience, type of source of information were significantly associated with knowledge, attitude and practice of HCWs toward COVID-19. So, the stakeholders must focus the training of HCWs for better practice of tackling with COVID-19. Special emphasis is required to HCWs of health centers.

## Supporting information

S1 File English version questionnaire. (DOCX)

S2 File Amharic version questionnaire. (DOCX)

S3 File Data set of the study. (XLSX)

S4 File Containing all the supporting tables. (DOCX)
