# Supplementary material for: Assessment of knowledge, attitude and practice toward COVID-19 and associated factors among health care workers in Silte Zone, Southern Ethiopia
Source: PLoS One. 2021 Oct 5;16(10):e0257058. doi: 10.1371/journal.pone.0257058 (PMC8491949; doi:10.1371/journal.pone.0257058)
Supplement: S1 File — (DOCX) [file pone.0257058.s001.docx]

1. **English version**

**Date of interview_____ Participant’s Unique ID_______Name of data collector___________**

Questionnaire to assess of knowledge, attitude and practice towards COVID-19 and associated factors among residents in Silte Zone, Southern Ethiopia, 2020

| **S. No.** | **Questions** | **Response Category** | **Skip/**  **Remark** |
| --- | --- | --- | --- |
| **1** | **Socio demographic and Economic characteristics** | | |
| 1.01 | What is your age  (In completed years)? | _____________years |  |
| 1.02 | What is your sex? | 1**.** Male 2. Female |  |
| 1.03 | What is your marital status | 1**.** Single  2. Married  3. Separated  4. Divorced  5. Windowed |  |
| 1.04 | What is your Religion? | 1. Protestant  2**.** Muslim  3. Orthodox 4. Catholic  5.Other (Specify)__________ |  |
| 1.05 | Where is your residence | 1**.** Urban 2. Rural |  |
| 1.06 | What is your ethnicity? | 1. Silte  2. Hadiya  3. Halaba  4. Gurage  5. Amhara  6.Other (specify)_______ |  |
| 07 | What is your occupation? | 1**.** House wife  2. Private employee  3. Government employee  4. Merchant  5. Farmer  6. Student  7. Daily laborer  8. No Job  9. Others (Specify)______ |  |
| 1.08 | What is your level of education | 1**.** No formal education  2. Primary (1-8) 3. Secondary (9-12)  4. College and above |  |
|  | What is your main source of information about COVID-19? | 1.Television 2.Socialmedia  3. Websites of hospital  4. Friends, relatives  5. Radio  6.Unheard  7. Others (Specify)______ |  |
| 1.09 | On average how many hours do you work per day? | ------------Hours |  |
| 1.10 | What is your monthly  family income in birr | **____________**birr |  |
|  |  |  |  |

| **02** | **Knowledge Related Questions** | | |  |
| --- | --- | --- | --- | --- |
| 2.01 | COVID 19 is viral disease | 1. Yes  2. No  3. I don’t Know | |  |
| 2.02 | COVID 19 can be transmitted through contact | 1. Yes  2. No  3. I don’t Know | |  |
| 2.03 | COVID 19 can transmit through stool | 1. Yes  2. No  3. I don’t Know | |  |
| 2.04 | COVID 19 can be transmitted through breast milk | 1. Yes  2. No  3. I don’t Know | |  |
| 2.05 | COVID 19 can be transmitted through contact with wild animals or eating raw meal | 1. Yes  2. No  3. I don’t Know | |  |
| 2.06 | The main reservoir of COVID 19 virus is bat | 1. Yes  2. No  3. I don’t Know | |  |
| 2.07 | Fever, cough , headache, SOB are major symptoms of COVID 19 | 1. Yes  2. No  3. I don’t Know | |  |
| 2.08 | COVID 19 is Severe among 80% of cases | 1. Yes  2. No  3. I don’t Know | |  |
| 2.09 | COVID 19 Patients from good economic status can be treated at home | 1. Yes  2. No  3. I don’t Know | |  |
| 2.10 | CKD, cancer, DM,HTN and Old people are at more risk for sever COVID 19 | 1. Yes  2. No  3. I don’t Know | |  |
| 2.11 | Currently best diagnosis for COVID 19 is Microscopy | 1. Yes  2. No  3. I don’t Know | |  |
| 2.12 | The first step if COVID-19 patient comes to your institution is treating the patient | 1. Yes  2. No  3. I don’t Know | |  |
| 2.13 | Currently major type of sample for COVID 19 is taken from saliva | 1. Yes  2. No  3. I don’t Know | |  |
| 2.14 | COVID 19 patients can be contagious before symptoms | 1. Yes  2. No  3. I don’t Know | |  |
| 2.15 | Mild cases can be cured within 2 weeks but severe cases can be cured with in 3 to 6 weeks | 1. Yes  2. No  3. I don’t Know | |  |
| 2.16 | Most severe complication of COVID 19 is pneumonia | 1. Yes  2. No  3. I don’t Know | |  |
| 2.17 | The best treatment for COVID 19 is antibiotics | -----------------Minutes | |  |
| 2.18 | Criteria for discharging COVID 19 patient are if no symptom within 14 days and least two negative results within 14 days. | 1. Yes  2. No  3. I don’t Know | |  |
| 2.19 | Mortality rate of COVID 19 is not more than 5 percent globally. | 1. Yes  2. No  3. I don’t Know | |  |
| 2.20 | COVID 19 does not have cure yet | 1. Yes  2. No  3. I don’t Know | |  |
| 21 | To prevent COVID 19 one should wash his/her hands with soap and water at least for 20 seconds | 1. Yes  2. No  3. I don’t Know | |  |
| 22 | Washing hands, holding the string, checking not thorn, checking inside and fitting with the nose are the first five steps of wearing facemask | 1. Yes  2. No  3. I don’t Know | |  |
| 23 | The incubation period for COVID 19 is 2-14 days | 1. Yes  2. No  3. I don’t Know | |  |
| 24 | COVID 19 is transmitted through droplets from mouth or nose | 1. Yes  2. No  3. I don’t Know | |  |
| 25 | COVID 19 cannot infect very young children | 1. Yes  2. No  3. I don’t Know | |  |
| 26 | COVID 19 can be transmitted through contaminated water | 1. Yes  2. No  3. I don’t Know | |  |
| 27 | COVID 19 may be severe on pregnant mothers | 1. Yes  2. No  3. I don’t Know | |  |
| 28 | COVID 19 can be transmitted through contaminated food | 1. Yes  2. No  3. I don’t Know | |  |
| 29 | COVID 19 can be transmitted through blood donation | 1. Yes  2. No  3. I don’t Know | |  |
| 30 | People with O blood group will not be infected with COVID 19 | 1. Yes  2. No  3. I don’t Know | |  |
| **03** | **Attitude Related Questions** | | |  |
| 3.01 | I am worry that I may be infected with COVID 19 | | 1. Strongly agree 2. Agree 3. Partially agree 4. Disagree 5. Strongly disagree |  |
| 3.02 | COVID 19 is severe diseases | | 1. Strongly agree 2. Agree 3. Partially agree 4. Disagree 5. Strongly disagree |  |
| 3.03 | I am worry that my family members may be infected | | 1. Strongly agree  2. Agree  3. Partially agree  4. Disagree  5. Strongly disagree |  |
| 3.04 | You are ready to get isolation treatment center if you are infected with COVID-19 | | 1. Strongly agree  2. Agree  3. Partially agree  4. Disagree  5. Strongly disagree |  |
| 3.05 | Proper hand washing can prevent COVID 19 | | 1. Strongly agree  2. Agree  3. Partially agree  4. Disagree  5. Strongly disagree |  |
| 3.06 | I am ready to take if COVID 19 vaccination is available | | 1. Strongly agree  2. Agree  3. Partially agree  4. Disagree  5. Strongly disagree |  |
| 3.07 | Relevant information about COVID 19 should be addressed for health professionals | | 1. Strongly agree  2. Agree  3. Partially agree  4. Disagree  5. Strongly disagree |  |
| 3.08 | To treat COVID 19 suspected case one should wear glove, mask, gown and eye goggle | | 1. Strongly agree  2. Agree  3. Partially agree  4. Disagree  5. Strongly disagree |  |
| 3.09 | implementing information from WHO is very important to prevent COVID 19 | | 1. Strongly agree  2. Agree  3. Partially agree  4. Disagree  5. Strongly disagree |  |
| 3.10 | Social distancing is crucial for prevention of COVID 19 | | 1. Strongly agree  2. Agree  3. Partially agree  4. Disagree  5. Strongly disagree |  |
| 3.11 | One should quarantine him/herself if he/she has symptoms of COVID 19 | | 1. Strongly agree  2. Agree  3. Partially agree  4. Disagree  5. Strongly disagree |  |
| 3.12 | Restricting transportation is very important to prevent COVID 19 | | 1. Strongly agree  2. Agree  3. Partially agree  4. Disagree  5. Strongly disagree |  |
| 3.13 | Hand washing with only water can prevent COVID 19 | | 1. Strongly agree  2. Agree  3. Partially agree  4. Disagree  5. Strongly disagree |  |
| 2.14 | Garlic, lemon, ginger and cumin oil are medicines for COVID 19 | | 1. Strongly agree  2. Agree  3. Partially agree  4. Disagree  5. Strongly disagree |  |
| 2.15 | COVID 19 is can be cured at home based treatment | | 1. Strongly agree  2. Agree  3. Partially agree  4. Disagree  5. Strongly disagree |  |
| 3.16 | Having precautions good to prevent COVID 19 | | 1. Strongly agree  2. Agree  3. Partially agree  4. Disagree  5. Strongly disagree |  |
| 3.17 | COVID 19 is curable diseases | | 1. Strongly agree  2. Agree  3. Partially agree  4. Disagree  5. Strongly disagree |  |
| 3.18 | Health professionals have good knowledge about COVID 19 | | 1. Strongly agree  2. Agree  3. Partially agree  4. Disagree  5. Strongly disagree |  |
| 3.19 | COVID 19 can be considered as curse from God/Allah | | 1. Strongly agree  2. Agree  3. Partially agree  4. Disagree  5. Strongly disagree |  |
| 3.20 | COVID 19 causes death in most of the cases | | 1. Strongly agree  2. Agree  3. Partially agree  4. Disagree  5. Strongly disagree |  |
| 3.21 | Ethiopia is at good standard of preventing and controlling COVID 19 | | 1. Strongly agree  2. Agree  3. Partially agree  4. Disagree  5. Strongly disagree |  |
| 3.22 | Black race is less affected with COVID 19 than White race | | 1. Strongly agree  2. Agree  3. Partially agree  4. Disagree  5. Strongly disagree |  |

| **04** | **Practice Related Factors** | | |
| --- | --- | --- | --- |
| 4.01 | Do you educate your patients about COVID 19? | 1. Yes  2. No  3. Sometimes |  |
| 4.02 | Do you wear face mask properly and always | 1. Yes  2. No  3. Sometimes |  |
| 4.03 | Are you careful to do not touch your mouth, nose and face? | 1. Yes  2. No  3. Sometimes |  |
| 4.04 | Do you cover your mouth and nose whenever you cough /sneeze? | 1. Yes  2. No  3. Sometimes |  |
| 4.05 | Do wash your hands with water and soap/sanitizer whenever you touch materials | 1. Yes  2. No  3. Sometimes |  |
| 4.06 | In recent days have you been in crowding area? | 1. Yes  2. No  3. Sometimes |  |
| 4.07 | Do you wash your hands with water and soap properly and frequently? | 1. Yes  2. No  3. Sometimes |  |
| 4.08 | Do you decide to stay at home if not obliged to get out? | 1. Yes  2. No  3. Sometimes |  |
| 4.09 | Have you quit consuming outside foods aiming to prevent COVID 19? | 1. Yes  2. No  3. Sometimes |  |
| 4.10 | Do you use traditional herbal medicines to prevent COVID 19? | 1. Yes  2. No  3. Sometimes |  |
| 4.11 | Do you use disinfection at your home to prevent COVID 19? | 1. Yes  2. No  3. Sometimes |  |
| 4.12 | \| Do you wash your hands after seeing every patient? \| \| --- \| | 1. Yes  2. No  3. Sometimes |  |
| 4.13 | Do wash your hands whenever you go back to your home just before getting home | 1. Yes  2. No  3. Sometimes |  |
| 4.14 | Have you reduced recreation aiming to prevent COVID 19? | 1. Yes  2. No  3. Sometimes |  |
| 4.15 | Do you eat balanced diet aiming to prevent COVID 19? | 1. Yes  2. No  3. Sometimes |  |
| 4.16 | Do you do physical exercise aiming to prevent COVID 19? | 1. Yes  2. No  3. Sometimes |  |

***The End!***

***Thank You for Your Cooperation!!!***
