# Supplementary material for: Assessment of knowledge, attitude and practice toward COVID-19 and associated factors among health care workers in Silte Zone, Southern Ethiopia
Source: PLoS One. 2021 Oct 5;16(10):e0257058. doi: 10.1371/journal.pone.0257058 (PMC8491949; doi:10.1371/journal.pone.0257058)
Supplement: S2 File — (DOCX) [file pone.0257058.s002.docx]

**የአማርኛ መጠይቅ**

**የመጠይቅ ቁጥር -------------------------------------- የመረጃ ሰባሳቢ ስም----------------------------- ቀን-------------**

| **ተ.ቁጥር** | **ጥያቄዎች** | **መልስ** | | | **ዝለል** | | | |
| --- | --- | --- | --- | --- | --- | --- | --- | --- |
| **1** | **ማህበራዊ ና ዲሞግራፊ ሁኔታዎችን ለመዳሰስ የሚረዱ መጠየቆችን በተመለከተ፡፡** | | | | | | | |
| 1.01 | ዕድሜዎ ስንት ነዉ? | **___________**ዓመት | | |  | | | |
| 1.02 | ፆታ | 1**.** ወንድ 2. ሴት | | |  | | | |
| 1.03 | የጋብቻ ሁኔታ? | 1/ያላገባ/ች 2/ያገባ/ች 3/የተለያዩ  4/የፈታ/ች 5/ የሞታባት/ችበት | | |  | | | |
| 1.04 | ሐይማኖት? | 1. ሙስሊም 2. ፕሮቴስታንት 3. አርቶዶክስ  4. ካቶሊክ 5. ሌላ (ይገለፅ)------- | | |  | | | |
| 1.05 | የመኖሪያ ቦታ? | 1/ ከተማ 2/ ገጠር | | |  | | | |
| 1.06 | የሚሰሩበት ተቋም | 1. ጤና ጣቢያ 2. የመጀመሪያ ደረጀ ሆስፒታል 3.ኮምፐርሄንሲቨ ሆስፒታል | | |  | | | |
| 1.07 | የት/ት ደረጀ | 1. ዲፕሎማ 2. ድግሪ 3. ማሰተርሰ 4. ሌላ---------------- | | |  | | | |
| 1.08 | ብሔረሰብ? | 1. ስልጤ 2. ሀዲያ 3. ካምባታ 4. ጉራጌ 5. አማራ 6. አላባ 7.ኦሮሞ 8.ሌላ (ይገለጽ)_______ | | |  | | | |
| 1.09 | ስለ ኮቪድ አብዛኛዉን እንፎርሜሽን የሚሰሙት ከየት ነዉ? | 1. ቴሌቪዢን 2. ማህባራዊ ሚዲያ 3 ከጤና ሚኒስቴር ዌብሳይት 4. ከጓደኛ ወይም ከዘመድ  5. ከሬዲዮ 6.ከየትም 7. ሌላ (ይገለፅ)______ | | |  | | | |
| 1.10 | ሙያዎት ምንድነዉ? | 1**.** ነርስ 2. ሚድዋይፍ 3. ላቦራቶሪ 4.ህብረተሰብ ጤና  5. ፋርማሲ 6. አንስቴዢያ 7. ሀኪም 8. ሌላ (ይገለፅ)____ | | |  | | | |
| 1.11 | የስራ ልምድዎ በአመት ስንት ነዉ? | _________አመት | | |  | | | |
| 1.12 | ስለ ኮቪድ-19 ስልጠና ወስደዉ ያዉቃሉ፡፡ | 1. አዎ 2. አይደልም | | |  | | | |
| 1.13 | በአማካይ በቀን ስንት ሰዓት ይሰራሉ? | ------------ሰዓት | | |  | | | |
| 1.14 | ወርሃዊ የቤተሰብ ገቢ ስንት ነዉ? | **____________**ብር | | |  | | | |
| **2** | **ከግንዛቤ ጋር ተያያዥነት ያለቸዉ ጥያቄዎች** | | | | | | | |
| 2.01 | ኮቪድ-19 በቨይረስ ምክንየት የሚመጣ በሽታ ነዉ? | | | 1. አዎ 2. አይደለም 3. አላዉቅም | | |  | |
| 2.02 | ኮቪድ-19 በሽታ ካለበት ሰዉ ጋር ንክክ በማድረግ ይተላለፋል? | | | 1. አዎ 2. አይደለም 3. አላዉቅም | | |  | |
| 2.03 | ኮቪድ-19 በሽታ ካለበት ሰዉ አይነ ምድር ንክክ በማድረግ ይተላለፋል? | | | 1. አዎ 2. አይደለም 3. አላዉቅም | | |  | |
| 2.04 | ኮቪድ-19 በሽታ ካለባት እናት ጡት ወተት ምክኒያት ይተላለፋል? | | | 1. አዎ 2. አይደለም 3. አላዉቅም | | |  | |
| 2.05 | ኮቪድ-19 በሽታ ከዱር እንስሳት ጋር ንክክ በማድረግ ወይም ጥሬ ስጋቸዉን በመመገብ ልታላለፍ ይችላል? | | | 1. አዎ 2. አይደለም 3. አላዉቅም | | |  | |
| 2.06 | ኮቪድ-19 በሽታን ልያስተላልፉ ከሚችሉ እንስሳት መካከል የሌሊት ወፍ ዋናኛ ናት፡፡ | | | 1. አዎ 2. አይደለም 3. አላዉቅም | | |  | |
| 2.07 | ትኩሳት፤ ሳል፤ ራስ ምታትና ለመተንፈስ መቸገር ዋና ዋና የኮቪድ-19 በሽታ ምልክቶች ናቸዉ? | | | 1. አዎ 2. አይደለም 3. አላዉቅም | | |  | |
| 2.08 | በኮቪድ-19 ከሚጠቁ ሰዎች መካከል ከባድ በሽታ ሊኖርባቸዉ የሚችለዉ 80 ከመቶ በሚሆኑት ላይ ነዉ፡፡ | | | 1. አዎ 2. አይደለም 3. አላዉቅም | | |  | |
| 2.09 | ጥሩ የኑሮ ደረጀ በላበቸዉ አካባቢዎች ቀለል ባለ ኮቪድ-19 የሚጠቁ ታማሚዎች ቤት ሆናዉ እንድታከሙ ይመከራል፡፡ | | | 1. አዎ 2. አይደለም 3. አላዉቅም | | |  | |
| 2.10 | ነባር የኩላሊት በሽታ፤ ካንሰር፤ የስኳር በሽታ፤ ደም ግፊት እና በእድሜ መግፋት ኮቪድ-19 በሽታን ከባድ ደረጀ እንደደርስ ከሚያደርጉ ሁኔታዎች መካካል ናቸዉ፡፡ | | | 1. አዎ 2. አይደለም 3. አላዉቅም | | |  | |
| 2.11 | ባሁኑ ወቅት ኮቪድ-19ን ለመመርመር በዋናነት የሚንጠቀመዉ ምርመራ የመይክሮስኮፒ ምርመራ ነዉ፡፡ | | | 1. አዎ 2. አይደለም 3. አላዉቅም | | |  | |
| 2.12 | አንድ በኮቪድ-19 መያዙ የተረጋገጠ ታማሚ አንተ ወደ ምትሰራበት ጤና ተቋም ቢመጣ ከሁሉ አስቀድሞ ማድረግ ያለብህ ታማሚዉን ቶሎ ብላህ ማከም ነዉ፡፡ | | | 1. አዎ 2. አይደለም 3. አላዉቅም | | |  | |
| 2.13 | ባሁኑ ወቅት ኮቪድ-19ን ለመመርመር በዋናነት የሚወሰደው ናሙና የምራቅ ናሙና ነዉ፡፡ | | | 1. አዎ 2. አይደለም 3. አላዉቅም | | |  | |
| 2.14 | በኮቪድ-19 የተጠቁ ሰዎች ምልክት ማሰየት ከመጀመራቸዉ በፊት በሽታዉን ለሌላ ሰዉ ማሳተላለፍ ይችላሉ? | | | 1. አዎ 2. አይደለም 3. አላዉቅም | | |  | |
| 2.15 | ቀለል በላ ኮቪድ-19 የተጠቁ ሰዎች በሁለት ሳምንታት ዉስጥ ሊሻለቸዉ ስችል ከበድ ባለ ኮቪድ-19 የተጠቁ ሰዎች ግን በስድስት ሳምንታት ውስጥ ሊሻለቸዉ ይችላል፡፡ | | | 1. አዎ 2. አይደለም 3. አላዉቅም | | |  | |
| 2.16 | በኮቪድ-19 የተያዙ ሰዎች በአብዘኛዉ የሚጎዱት በሳንበ ምች ነዉ፡፡ | | | 1. አዎ 2. አይደለም 3. አላዉቅም | | |  | |
| 2.17 | ለኮቪድ-19 ካሉ የህክምና አመራጮች ውስጥ የተሻለ ህክምና አንቲበዮቲክስ ነዉ፡፡ | | | 1. አዎ 2. አይደለም 3. አላዉቅም | | |  | |
| 2.18 | አንድ በኮቪድ-19 የተጠቀ ሰዉ ህክምና ማዕከል ከገባ በኋላ ለመዉጣት መስፈርቶች መካከል፤ በ14 ቀን ዉስጥ ምንም አይነት ምልክት አለማሰየትና ቢያንስ 2 ናሙና ተወስዶ ዉጤቱ ኔጋቲቨ መሆን ናቸዉ፡፡ | | | 1. አዎ 2. አይደለም 3. አላዉቅም | | |  | |
| 2.19 | እስካሁን ባለዉ አለም አቀፍ መረጀ የኮቪድ-19 የመግደል አቅም 5 ከመቶ የዘላለ አይደላም፡፡ | | | 1. አዎ 2. አይደለም 3. አላዉቅም | | |  | |
| 2.20 | አለም እስካሁን የኮቪድ-19 ፍቱን መድሃኒት አላገኘችም፡፡ | | | 1. አዎ 2. አይደለም 3. አላዉቅም | | |  | |
| 2.21 | ኮቪድ-19ን ለመከላከል አንድ ሰዉ ቢያንስ ለ20 ሰከንድ እጅን መታጠብ አለበት፡፡ | | | 1. አዎ 2. አይደለም 3. አላዉቅም | | |  | |
| 2.22 | እጅን ባግባቡ መታጠብ፤የጭንብሉን ማሳሪያ ብቻ መያዝ፤ ያልተቀደደ መሁኑን መረጋገጥ፤ ውስጠኛዉንና ዉጨኛዉን ገፅ መለየትና አፍንጫዉን በትክክል ማጣበቅ ትክክለኛ የፍት ጭንብል አደረረግ ውስጥ የመጀመሪያ አምስት ቅድም ተከተሎች ናቸዉ፡፡ | | | 1. አዎ 2. አይደለም 3. አላዉቅም | | |  | |
| 2.23 | የኮቪድ-19 ምልክት የሚታየዉና የሚጀምረዉ በመጀመሪያ 2-14 ቀናት ዉስጥ ነዉ፡፡ | | | 1. አዎ 2. አይደለም 3. አላዉቅም | | |  | |
| 2.24 | ኮቪድ-19 ከታማሚ አፍና አፍንጫ በሚወጡ ብናኞችና በንክኪ የሚተላለፍ በሽታ ነዉ፡፡ | | | 1. አዎ 2. አይደለም 3. አላዉቅም | | |  | |
| 2.25 | ኮቪድ-19 በእድሜ በጣም ትንሽ የሆኑ ህፃናትን አያጠቃም፡፡ | | | 1. አዎ 2. አይደለም 3. አላዉቅም | | |  | |
| 2.26 | ኮቪድ-19 በተበከለ ዉሃ ሊተላለፍ ይችላል፡፡ | | | 1. አዎ 2. አይደለም 3. አላዉቅም | | |  | |
| 2.27 | ኮቪድ-19 በነብሰ ጡር እናቶች የበለጠ ይከፋል፡፡ | | | 1. አዎ 2. አይደለም 3. አላዉቅም | | |  | |
| 2.28 | ኮቪድ-19 በምግብ ምክንያት ሊተላለፍ ይችላል፡፡ | | | 1. አዎ 2. አይደለም 3. አላዉቅም | | |  | |
| 2.29 | ኮቪድ-19 በደም ልገሰ ምክንያት ሊተላለፍ ይችላል፡፡ | | | 1. አዎ 2. አይደለም 3. አላዉቅም | | |  | |
| 2.30 | ኦ(O) የደም አይነት ያለቸዉን ሰዎች ኮቪድ-19 አያጠቀቸዉም፡፡ | | | 1. አዎ 2. አይደለም 3. አላዉቅም | | |  | |
| 3 | **ስለ ኮቪድ-19 ያሎትን አመለካከት የሚለኩ ጥያቄዎች** | | | | | | | |
| 3.01 | ኮቪድ-19 በሽታ ሊይዘኝ ይችላል ብዬ እጨነቃለዉ፡፡ | 1. በጣም እስማማለሁ 2. እስማማለሁ 3. በከፊል እስማማለሁ  4. አልስማማም 5. በጣም አልስማማም | | | |  | | |
| 3.02 | ቤተሰቦቼ በኮቪድ-19 ልጠቁ ይችላሉ ብዬ እጨነቃለዉ፡፡ | 1. በጣም እስማማለሁ 2. እስማማለሁ 3. በከፊል እስማማለሁ  4. አልስማማም 5. በጣም አልስማማም | | | |  | | |
| 3.03 | እርስዎ በኮቪድ-19 በሽታ ቢያዙ ለይቶ ማቆያ ገብተዉ ለመታካም ፍቀደኛ ነኝ፡፡ | 1. በጣም እስማማለሁ 2. እስማማለሁ 3. በከፊል እስማማለሁ  4. አልስማማም 5. በጣም አልስማማም | | | |  | | |
| 3.04 | ባግባቡ እጅ መታጠብ ኮቪድ-19 በሸታን ሊከላከል ይችላል? | 1. በጣም እስማማለሁ 2. እስማማለሁ 3. በከፊል እስማማለሁ  4. አልስማማም 5. በጣም አልስማማም | | | |  | | |
| 3.05 | የኮቪድ-19 ክትባት ቢገኝ ለመውሰድ ዝግጁ ነኝ፡፡ | 1. በጣም እስማማለሁ 2. እስማማለሁ 3. በከፊል እስማማለሁ  4. አልስማማም 5. በጣም አልስማማም | | | |  | | |
| 3.06 | በአለም አቀፍ ጤና ድርጅቶች የሚታላለፉ መልዕክቶችን ባግባቡ መተግበር የኮቪድ-19ን ለመከላከል ይረዳል፡፡ | 1. በጣም እስማማለሁ 2. እስማማለሁ 3. በከፊል እስማማለሁ  4. አልስማማም 5. በጣም አልስማማም | | | |  | | |
| 3.07 | ስለኮቪድ-19 አግባበዊ የሆኑ መልዕክቶች ለጤና በላሙያዎች በየግዜዉ መድረስ አላበቸዉ፡፡ | 1. በጣም እስማማለሁ 2. እስማማለሁ 3. በከፊል እስማማለሁ  4. አልስማማም 5. በጣም አልስማማም | | | |  | | |
| 3.08 | በኮቪድ-19 የሚጠረጠር ሰዉን ለማከም ጋዉን፤ማስክ፤ግላቭና አይጉግል ማድረግ ግድ ነዉ፡፡ | 1. በጣም እስማማለሁ 2. እስማማለሁ 3. በከፊል እስማማለሁ  4. አልስማማም 5. በጣም አልስማማም | | | |  | | |
| 3.09 | ማህባራዊ ርቀትን መጠበቅ ኮቪድ-19ን ለመከለካል ወሳኝ ነዉ፡፡ | 1. በጣም እስማማለሁ 2. እስማማለሁ 3. በከፊል እስማማለሁ  4. አልስማማም 5. በጣም አልስማማም | | | |  | | |
| 3.10 | አንድ ግለሰብ ኮቪድ-19ን የሚመስሉ ምልክቶችን ካያ እራሱን ማግላል አለበት፡፡ | 1. በጣም እስማማለሁ 2. እስማማለሁ 3. በከፊል እስማማለሁ  4. አልስማማም 5. በጣም አልስማማም | | | |  | | |
| 3.11 | ማህበራዊ ማጓጓዠን ማስቆም/መገደብ ኮቪድ-19 ለመከላከል በጣም ጣቃሚ ነዉ፡፡ | 1. በጣም እስማማለሁ 2. እስማማለሁ 3. በከፊል እስማማለሁ  4. አልስማማም 5. በጣም አልስማማም | | | |  | | |
| 3.12 | እጅን በዉሃ ብቻ መታጠብ ኮቪድ-19ን ይከላከላል? | 1. በጣም እስማማለሁ 2. እስማማለሁ 3. በከፊል እስማማለሁ  4. አልስማማም 5. በጣም አልስማማም5. በጣም አልስማማም | | | |  | | |
| 3.13 | ነጭ ሽንኩርት፤ሎሚ፤ ጤና አዳምና ጥቁር አዝሙትን የመሳሰሉ ባህላዊ መድሃኒቶች ለኮቪድ-19 መድሃኒት ናቸዉ፡፡ | 1. በጣም እስማማለሁ 2. እስማማለሁ 3. በከፊል እስማማለሁ  4. አልስማማም 5. በጣም አልስማማም | | | |  | | |
| 3.14 | ኮቪድ-19 ከባድ በሽታ ነዉ፡፡ | 1. በጣም እስማማለሁ 2. እስማማለሁ 3. በከፊል እስማማለሁ  4. አልስማማም 5. በጣም አልስማማም | | | |  | | |
| 3.15 | ኮቪድ-19 በቤት ውስጥ ህክምና ልድን ይችላል፡፡ | 1. በጣም እስማማለሁ 2. እስማማለሁ 3. በከፊል እስማማለሁ  4. አልስማማም 5. በጣም አልስማማም | | | |  | | |
| 3.16 | ጥንቃቄዎችን ማድረግ ኮቪድ-19ን ይከላከላል፡፡ | 1. በጣም እስማማለሁ 2. እስማማለሁ 3. በከፊል እስማማለሁ  4. አልስማማም 5. በጣም አልስማማም | | | |  | | |
| 3.17 | ኮቪድ-19 ሙሉ በሙሉ የሚድን በሽታ ነው፡፡ | 1. በጣም እስማማለሁ 2. እስማማለሁ 3. በከፊል እስማማለሁ  4. አልስማማም 5. በጣም አልስማማም | | | |  | | |
| 3.18 | የጤና ባለሙያዎች ስለኮቪድ-19 በቂ ግንዛቤ አላቸዉ፡፡ | 1. በጣም እስማማለሁ 2. እስማማለሁ 3. በከፊል እስማማለሁ  4. አልስማማም 5. በጣም አልስማማም | | | |  | | |
| 3.19 | ኮቪድ-19 የፈጣሪ ቁጣ/ቅጣት ነዉ፡፡ | 1. በጣም እስማማለሁ 2. እስማማለሁ 3. በከፊል እስማማለሁ  4. አልስማማም 5. በጣም አልስማማም | | | |  | | |
| 3.20 | ኮቪድ-19 በአብዛኛዉ ሰዉ ሞትን ያስከትላል፡፡ | 1. በጣም እስማማለሁ 2. እስማማለሁ 3. በከፊል እስማማለሁ  4. አልስማማም 5. በጣም አልስማማም | | | |  | | |
| 2.21 | ኢትዮጵያ ኮቪድ-19 በመከላከል ረገድ በጥሩ አቋም ላይ ትገኛለች፡፡ | 1. በጣም እስማማለሁ 2. እስማማለሁ 3. በከፊል እስማማለሁ  4. አልስማማም 5. በጣም አልስማማም | | | |  | | |
| 3.22 | ጥቁር ዘር መሆን በኮቪድ-19 የመያዝ እድልን ይቀንሳል፡፡ | 1. በጣም እስማማለሁ 2. እስማማለሁ 3. በከፊል እስማማለሁ  4. አልስማማም 5. በጣም አልስማማም | | | |  | | |
| 4 | **ስለ ኮቪድ-19 መከላከል ተግባሮችን በተመለከተ የተዘጋጁ ጥያቄዎች** | | | | | | | |
| 4.01 | ታካሚዎን ስለኮቪድ-19 የስተምራሉ? | | 1. አዎ 2. አይደለም 3. አንዳንዴ | | | | |  |
| 4.02 | የፍት ጭንበልን ባግባቡና ሁልግዜ ይጠቀማሉ? | | 1. አዎ 2. አይደለም 3. አንዳንዴ | | | | |  |
| 4.03 | በተቻለ መጠን አፍ፤አፍንጫዎንና ፍትዎን ለለመንካት ጥንቃቄ ያደርጋሉ? | | 1. አዎ 2. አይደለም 3. አንዳንዴ | | | | |  |
| 4.04 | በሚያስሉበትና በሚያስነጥሱበት ግዜ አፍና አፍንጫዎትን ይሸፍናሉ? | | 1. አዎ 2. አይደለም 3. አንዳንዴ | | | | |  |
| 4.05 | እጅዎን በዉሃና በሳሙና በተደጋገሚ ይታጠባሉ? | | 1. አዎ 2. አይደለም 3. አንዳንዴ | | | | |  |
| 4.06 | በቅርብ ቀናት ውስጥ ሰዉ የሚበዘበትን አከባቢ ሄደዉ ያዉቃሉ? | | 1. አዎ 2. አይደለም 3. አንዳንዴ | | | | |  |
| 4.07 | በስራ ቦታ የተላያዩ እቃዎችን ሲነኩ እጅዎን በዉሃና በሳሙና ይታጠባሉ ወይም በሳኒታይዘር ያፀዳሉ? | | 1. አዎ 2. አይደለም 3. አንዳንዴ | | | | |  |
| 4.08 | ግድ ካልሆና በስተቀር እቤት ውስጥ ለመቆየት ይወስናሉ? | | 1. አዎ 2. አይደለም 3. አንዳንዴ | | | | |  |
| 4.09 | ኮቪድ-19 ለመከላከል እንድረዳዎት ብለዉ የዉጪ ምግብ መመገብ ትተዋል? | | 1. አዎ 2. አይደለም 3. አንዳንዴ | | | | |  |
| 4.10 | ኮቪድ-19 ለመከላከል እንዲረዳዎት ቤት ዉስጥ ለባህላዊ ህክምና የሚሆኑ መድሃኒቶችን/ምግቦችን ይጠቀማሉ? | | 1. አዎ 2. አይደለም 3. አንዳንዴ | | | | |  |
| 4.11 | ኮቪድ-19 ለመከላከል እንዲረዳ ቤት ዉስጥ ፀረ ተዋህሲያን ዉህዶችን ይጠቀማሉ? | | 1. አዎ 2. አይደለም 3. አንዳንዴ | | | | |  |
| 4.12 | አንድ ታካሚ ካዩ በኋላ ሌላዉን ከማየትዎ በፊት እጅ ይታጠባሉ? | | 1. አዎ 2. አይደለም 3. አንዳንዴ | | | | |  |
| 4.13 | ስራ ውለዉ ስመለሱ ቤት ውስጥ ከማንም ሰዉ ከመገናኛትዎ በፊት እጅዎን በሳሙናና በዉሃ ይታጠባሉ? | | 1. አዎ 2. አይደለም 3. አንዳንዴ | | | | |  |
| 4.14 | ኮቪድ-19 ለመከላከል እንዲረዳዎት መዝናኛዎችን ቀንሰዋል? | | 1. አዎ 2. አይደለም 3. አንዳንዴ | | | | |  |
| 4.15 | ኮቪድ-19ን ለመከላከል እንደሚረደዎት በማሰብ የተመጣጠና ምግብ ይመገባሉ? | | 1. አዎ 2. አይደለም 3. አንዳንዴ | | | | |  |
| 4.16 | ኮቪድ-19ን ለመከላከል እንደሚረደዎት በማሰብ የአካል ብቃት እንቅስቃሴ ያደርጋሉ? | | 1. አዎ 2. አይደለም 3. አንዳንዴ | | | | |  |

**እስካሁን ስላደረጉልኝ ትብብር ከልብ የመነጫ ምስጋናዬን አቀርባለዉ፡፡**
