# Supplementary material for: Assessment of knowledge, attitude and practice toward COVID-19 and associated factors among health care workers in Silte Zone, Southern Ethiopia
Source: PLoS One. 2021 Oct 5;16(10):e0257058. doi: 10.1371/journal.pone.0257058 (PMC8491949; doi:10.1371/journal.pone.0257058)
Supplement: S4 File — (DOCX) [file pone.0257058.s004.docx]

**Table 1: Response for knowledge Questions** (**Yes=Yes; No=no/I don’t know) among health care workers in Silte zone, southern Ethiopia, 2020.**

|  | **Question (n=379)** | **Response** | |
| --- | --- | --- | --- |
|  |  | **Yes (%)** | **No (%)** |
|  | COVID 19 is viral disease | 370 (97.6) | 9 (2.4) |
|  | COVID 19 can be transmitted through contact | 367 (96.8) | 12 (3.2) |
|  | COVID 19 can transmit through stool | 160 (42.2) | 219 (57.8) |
|  | COVID 19 can be transmitted through breast milk | 177 (46.7) | 202 (53.3) |
|  | COVID 19 can be transmitted through contact with wild animals or eating raw meal | 66 (17.4) | 313 (82.6) |
|  | The main reservoir of COVID 19 virus is bat | 226 (59.6) | 153 (40.4) |
|  | Fever, cough , headache, SOB are major symptoms of COVID 19 | 355 (93.7) | 24 (6.3) |
|  | COVID 19 is Severe among 80% of cases | 121 (31.9) | 258 (68.1) |
|  | COVID 19 Patients from good economic status can be treated at home | 87 (23) | 292 (77.0) |
|  | CKD, cancer, DM,HTN and Old people are at more risk for sever COVID 19 | 336 (88.7) | 43 (11.3) |
|  | Currently best diagnosis for COVID 19 is Microscopy | 196 (51.7) | 183 (48.3) |
|  | The first step if COVID-19 patient comes to your institution is treating the patient | 195 (51.5) | 184 (48.5) |
|  | Currently major type of sample for COVID 19 is taken from saliva | 135 (35.6) | 244 (64.4) |
|  | COVID 19 patients can be contagious before symptoms | 335 (88.4) | 44 (11.6) |
|  | Mild cases can be cured within 2 weeks but severe cases can be cured with in 3 to 6 weeks | 276 (72.8) | 103 (27.2) |
|  | Most severe complication of COVID 19 is pneumonia | 303 (79.9) | 76 (20.1) |
|  | The best treatment for COVID 19 is antibiotics | 168 (44.3) | 211 (55.7) |
|  | Criteria for discharging COVID 19 patient are if no symptom within 14 days and least two negative results within 14 days. | 321 (84.7) | 58 (15.3) |
|  | Mortality rate of COVID 19 is not more than 5 percent globally. | 226 (59.6) | 153 (40.4) |
|  | COVID 19 does not have cure yet | 349 (92.1) | 30 (7.9) |
|  | To prevent COVID 19 one should wash his/her hands with soap and water at least for 20 seconds | 349 (92.1) | 30 (7.9) |
|  | Washing hands, holding the string, checking not thorn, checking inside and fitting with the nose are the first five steps of wearing facemask | 334 (88.1) | 45 (11.9) |
|  | The incubation period for COVID 19 is 2-14 days | 335 (88.4) | 44 (11.6) |
|  | COVID 19 is transmitted through droplets from mouth or nose | 316 (83.4) | 63 (16.6) |
|  | COVID 19 cannot infect very young children | 254 (67.0) | 125 (33.0) |
|  | COVID 19 can be transmitted through contaminated water | 232 (61.2) | 147 (38.8) |
|  | COVID 19 may be severe on pregnant mothers | 194 (51.2) | 185 (48.8) |
|  | COVID 19 can be transmitted through contaminated food | 216 (57.0) | 163 (43.0) |
|  | COVID 19 can be transmitted through blood donation | 192 (50.7) | 187 (49.30) |
|  | People with O blood group will not be infected with COVID 19 | 260 (68.6) | 119 (31.4) |

**Table 2: Response for attitude questions (Agree= strongly agree/agree; disagree=undecided/disagree/strongly disagree) among health care workers in Silte zone, southern Ethiopia, 2020.**

|  | **Question (n=379)** | **Response** | |
| --- | --- | --- | --- |
|  |  | **Agree (%)** | **Disagree (%)** |
|  | I am worry that I may be infected with COVID 19 | 333 (87.9) | 46 (12.1) |
|  | COVID 19 is severe diseases | 328 (86.5) | 51 (13.5) |
|  | I am worry that my family members may be infected | 352 (92.9)) | 27(7.1) |
|  | You are ready to get isolation treatment center if you are infected with COVID-19 | 350 (92.3) | 29 (7.7) |
|  | Proper hand washing can prevent COVID 19 | 341 (90.0) | 38 (10.0) |
|  | I am ready to take if COVID 19 vaccination is available | 348 (91.8) | 31 (8.2) |
|  | Relevant information about COVID 19 should be addressed for health professionals | 336 (88.7) | 43 (11.3) |
|  | To treat COVID 19 suspected case one should wear glove, mask, gown and eye goggle | 333 (87.9) | 46 (12.1) |
|  | implementing information from WHO is very important to prevent COVID 19 | 338 (89.2) | 41 (10.8) |
|  | Social distancing is crucial for prevention of COVID 19 | 324 (85.5) | 55 (14.5) |
|  | One should quarantine him/herself if he/she has symptoms of COVID 19 | 320 (84.4) | 59 (15.6) |
|  | Restricting transportation is very important to prevent COVID 19 | 306 (80.7) | 73 (19.3) |
|  | Hand washing with only water can prevent COVID 19 | 201 (53.0) | 178 (47.0) |
|  | Garlic, lemon, ginger and cumin oil are medicines for COVID 19 | 221 (58.3) | 158 (41.7) |
|  | COVID 19 is can be cured at home based treatment | 289 (76.3) | 90 (23.7) |
|  | Having precautions good to prevent COVID 19 | 331 (87.3) | 48 (12.7) |
|  | COVID 19 is curable diseases | 311 (82.1) | 68 (17.9) |
|  | Health professionals have good knowledge about COVID 19 | 305 (80.5) | 74 (19.5) |
|  | COVID 19 can be considered as curse from God/Allah | 304 (80.2) | 75 (19.8) |
|  | COVID 19 causes death in most of the cases | 273 (72.0) | 106 (28.0) |
|  | Ethiopia is at good standard of preventing and controlling COVID 19 | 231 (60.9) | 148 (39.1) |
|  | Black race is less affected with COVID 19 than White race | 183 (48.3) | 196 (51.7) |

**Table 3: Practice related questions (Yes=Yes; No= No/Sometimes) among health among health care workers in Silte zone, southern Ethiopia, 2020.**

|  | **Question (n=379)** | **Response** | |
| --- | --- | --- | --- |
|  |  | **Yes (%)** | **No (%)** |
|  | Do you educate your patients about COVID 19? | 297 (78.4) | 82 (21.6) |
|  | Do you wear face mask properly and always | 300 (79.2) | 79 (20.8) |
|  | Are you careful to do not touch your mouth, nose and face? | 321 (84.7) | 58 (15.3) |
|  | Do you cover your mouth and nose whenever you cough /sneeze? | 326 (86.0) | 53 (14.0) |
|  | Do wash your hands with water and soap/sanitizer whenever you touch materials | 307 (81.0) | 72 (19.0) |
|  | In recent days have you been in crowding area? | 103 (27.2) | 276 (72.8) |
|  | Do you wash your hands with water and soap properly and frequently? | 312 (82.3) | 67 (17.7) |
|  | Do you decide to stay at home if not obliged to get out? | 276 (72.8) | 103 (27.2) |
|  | Have you quit consuming outside foods aiming to prevent COVID 19? | 236 (62.3) | 143 (37.7) |
|  | Do you use traditional herbal medicines to prevent COVID 19? | 167 (44.1) | 212 (55.9) |
|  | Do you use disinfection at your home to prevent COVID 19? | 216 (57.0) | 163 (43.0) |
|  | \| Do you wash your hands after seeing every patient? \| \| --- \| | 275 (72.6) | 104 (27.4) |
|  | Do wash your hands whenever you go back to your home just before getting home | 310 (81.8) | 69 (18.2) |
|  | Have you reduced recreation aiming to prevent COVID 19? | 304 (80.2) | 75 (19.8) |
|  | Do you eat balanced diet aiming to prevent COVID 19? | 254 (67.0) | 125 (33.0) |
|  | Do you do physical exercise aiming to prevent COVID 19? | 252 (66.5) | 127 (33.5) |
